# Relationship between dietary acid-base load and non-insulin-based resistance measures in patients with chronic kidney disease

**DOI:** 10.3389/fendo.2025.1589528

**Published:** 2025-06-20

**Authors:** Hui Huang, Qian Wang, Ruimin Zhang, Fang Liu, Yue Niu, Yayong Luo, Junqian Wang, Shuang Li, Zhengchun Tang, Xueying Cao, Xiaolong Wang, Jian Yang, Sha Luo, Weizhu Deng, Weiguang Zhang, Ying Zheng, Yong Wang, Li Zhang, Guangyan Cai, Xiangmei Chen, Zheyi Dong

**Affiliations:** ^1^ School of Clinical Medicine, Chengdu University of Traditional Chinese Medicine, Chengdu, China; ^2^ Department of Nephrology, First Medical Center of Chinese PLA General Hospital, State Key Laboratory of Kidney Diseases, National Clinical Research Center for Kidney Diseases, Beijing Key Laboratory of Medical Devices and Integrated Traditional Chinese and Western Drug Development for Severe Kidney Diseases, Beijing Key Laboratory of Digital Intelligent TCM for the Prevention and Treatment of Pan-vascular Disease, Key Disciplines of National Administration of Traditional Chinese Medicine (zyyzdxk-2023310), Beijing, China; ^3^ School of Clinical Medicine, Guangdong Pharmaceutical University, Guangzhou, China

**Keywords:** chronic kidney disease, dietary acid-base load, triglyceride glucose-body mass index, metabolic score for insulin resistance, insulin resistance

## Abstract

This study explored the associations between triglyceride glucose (TyG), TyG with body mass index (TyG-BMI), triglyceride-to-high-density lipoprotein cholesterol (TG/HDL-C) ratio, and metabolic score for insulin resistance (METS-IR) and the effects of dietary acid-base load in patients with chronic kidney disease (CKD).

**Methods:**

A total of 288 patients with CKD were included in this study. Four non-insulin-based insulin resistance (IR) markers were used to assess IR levels in patients with CKD; dietary intake – 24-h dietary recall; and diet-based acidity – potential renal acid load (PRAL), net endogenous acid production (NEAP), and dietary acid load (DAL). Multiple linear regression analysis correlated dietary acid-base load and non-insulin-based IR markers.

**Results:**

Spearman’s correlation indicated DAL was significantly associated with TyG-BMI (r = 0.251, P < 0.001) and METS-IR (r = 0.274, P < 0.001), but weakly correlated with the TG/HDL-C ratio (r = 0.14, P = 0.018). After adjusting for sex, age, energy, hypertension (HTN), diabetes, and estimated glomerular filtration rate, multiple linear regression analysis showed that DAL was associated with TyG-BMI (β = 0.336; P = 0.008) and METS-IR (β = 0.091; P = 0.007).

**Conclusions:**

Patients with the highest DAL scores had the highest TyG-BMI, TyG, TG/HDL-C ratio, and METS-IR. After adjusting for confounders, there was a significant positive association between DAL and TyG-BMI and METS-IR.

## 1 Introduction

The worldwide prevalence of chronic kidney disease (CKD) is around 9.5%, or approximately 850 million cases (1), while that in China is 8.2% (2). The high prevalence and long course of CKD have caused a huge economic burden to patients and society, as well as presented a great challenge to the medical profession. Moreover, many patients do not undergo renal replacement therapy. Prompt diagnosis and treatment are critical for patient outcomes in CKD, and can even delay disease progression and reduce complications. Studies have reported that CKD patients have many risk elements, including type 2 diabetes mellitus (T2DM), metabolic syndrome, obesity, hypertension (HTN), and dyslipidemia (2–4). These conditions are intricately linked to insulin resistance (IR), which exerts detrimental effects on the kidney by inducing inflammation, oxidative stress, and endothelial dysfunction (5–7). Therefore, the early recognition and effective management of IR can prevent or delay the development of CKD and its associated complications. The hyper insulinemic-euglycemic clamp technique is the benchmark for evaluating IR. It does this by infusing insulin to reach specific plasma levels and glucose to keep blood sugar at fasting or post-meal concentrations (8). However, it is impractical for clinical use because it requires insulin measurements and invasive methods. Therefore, four non-insulin-based IR markers – triglyceride and glucose index (TyG), triglyceride and glucose index with body mass index (TyG-BMI), triglyceride-to-high-density lipoprotein cholesterol (TG/HDL-C) ratio, and metabolic score for insulin resistance (METS-IR) – were utilized to assess the IR levels in patients with CKD, as in previous studies (9, 10).

Healthy dietary habits such as the HTN control and Mediterranean diets protect against the development of CKD and albuminuria (11). Additionally, a healthy acid-base balance is essential for maintaining metabolic health. Potential renal acid load (PRAL) (12), net endogenous acid production (NEAP) (13), and dietary acid load (DAL) (14) are commonly used measures to assess the potential acid load produced by the diet. Both the PRAL and NEAP scores are based on the intake of protein and micronutrients, and the DAL score is calculated using the PRAL score and body surface area. There is evidence that a high dietary acid load is linked to a high prevalence of CKD and impaired renal function (15–17).Researches have shown that a diet characterized by a high acid load correlates with metabolic abnormalities, predisposing individuals to IR (18), T2DM (19, 20), HTN (21), and metabolic syndrome (22).

The purpose of this study was to investigate the relationship between dietary acid and base load and four non-insulin-based IR markers in patients with CKD.

## 2 Materials and methods

### 2.1 Study population

Data were collected from patients with CKD who were hospitalized at the Chinese People’s Liberation Army general Hospital (PLAGH) from March 2022 to July 2023. Inclusion criteria: (1) age ≥ 18 years, and (2) diagnosis of CKD according to the 2024 Kidney Disease Improving Global Outcomes Clinical Practice Guidelines (23). Exclusion criteria: (1) history of severe infection, (2) acute and severe diseases, (3) pregnancy or lactation, (4) malignant tumors, (5) missing data of medical history or clinical examination results, and (6) incomplete dietary intake data and/or extreme energy intake reporting (> 4000 kcal or < 600 kcal). Ultimately, 288 non-dialysis patients with CKD were included.

### 2.2 Clinical data

The patients’ demographic and clinical characteristics (sex, height, weight, and age) and medical history (present illness, nephropathy, HTN, and diabetes) were recorded. Laboratory parameters evaluated including: white blood cell count (WBC), hemoglobin, total protein, albumin, haptoglobin, prealbumin, blood urea nitrogen (BUN), serum creatinine, estimated glomerular filtration rate (eGFR) (calculated using the CKD Epidemiology Collaboration formula), serum cystatin C, 24-h urinary protein, serum uric acid, total cholesterol, triglycerides, fasting blood glucose (FBG), serum calcium, potassium, phosphorus, high-density lipoprotein cholesterol(HDL-C), and low-density lipoprotein cholesterol (LDL-C).

### 2.3 Non-insulin-based IR indices

The non-insulin-based IR measures utilized were the TyG, TyG-BMI, TG/HDL-C, and METS-IR, calculated using the following formulas (10):


TyG=ln[TG(mg/dL)×FBG(mg/dL)/2];

TyG−BMI=TyG index×BMI;

TG/HDL−C=TG(mg/dL)/HDL−C(mg/dL);

$$\text{and METS}-\text{IR}=(\text{ln}[2\times \text{FBG}(\text{mg}/\text{dL})+\text{TG}(\text{mg}/\text{dL})]\times \text{BMI}(\text{kg}/{\text{m}}^{2})/\text{ln}[\text{HDL}-\text{C}(\text{mg}/\text{dL})]).$$

### 2.4 Dietary assessment

During the 24-h dietary review, the researchers directly asked the patients about their food consumption on the preceding day, evaluating the variety and quantities of food using tools such as food pictures or models. Nutrient intake was calculated according to the Chinese Dietary Guidelines (2022 edition) (24) and Chinese Dietary Reference Intakes (2013 edition) (25). Food and nutrient intake were then adjusted using the residual energy method (26).

Common indicators of dietary acid-base load include the NEAP, PRAL, calculated based on the dietary intake of proteins and minerals, and DAL, calculated using dietary protein, phosphorus, potassium, calcium, magnesium, height, and weight, as follows:

PRAL (mmol/d) (12) = 0.49 × protein (g/day) + 0.037 × phosphorus (mg/day) - 0.021 × potassium (mg/day) − 0.026 × magnesium (mg/day) - 0.013 × calcium (mg/day);
NEAP (mEq/d) (13) = 54.5 × [protein(g/day) / potassium intake (mEq/day)] - 10.2;
and DAL (mmol/d) (14) = PRAL + (body surface area [m^2^] × 41[mEq/d] / 1.73 m²), with body surface area (27, 28) = 0.007184 × height (cm) ^ 0.725 × weight (kg) ^ 0.425.

### 2.5 Statistical analysis

SPSS 26.0 statistical software was used for data analysis (SPSS Inc., Chicago, IL, USA). Normally distributed data were expressed as mean ± standard, while non-normally distributed data were expressed as medians with interquartile ranges. Differences between DAL tertiles were compared, and measurement data with a normal distribution and homogeneity of variance were compared between the groups using one-way analysis of variance. A non-parametric test was used to compare groups if the homogeneity of variance was not satisfied. Count variables were expressed as frequencies and percentages, and were analyzed using chi-squared or Fisher’s exact tests. Spearman’s correlation analysis was used to analyze the correlation between dietary acid and base load (including PRAL, NEAP, and DAL) and the non-insulin-based IR predictor indices, and multiple linear regression was used to assess the adjusted effects of variables affecting the non-insulin-based IR predictor indices. All *P* (or *P*-trends) were two-tailed, and statistical significance was set at *P* (or *p*-trend) < 0.05.

## 3 Results

**Figure 1:**
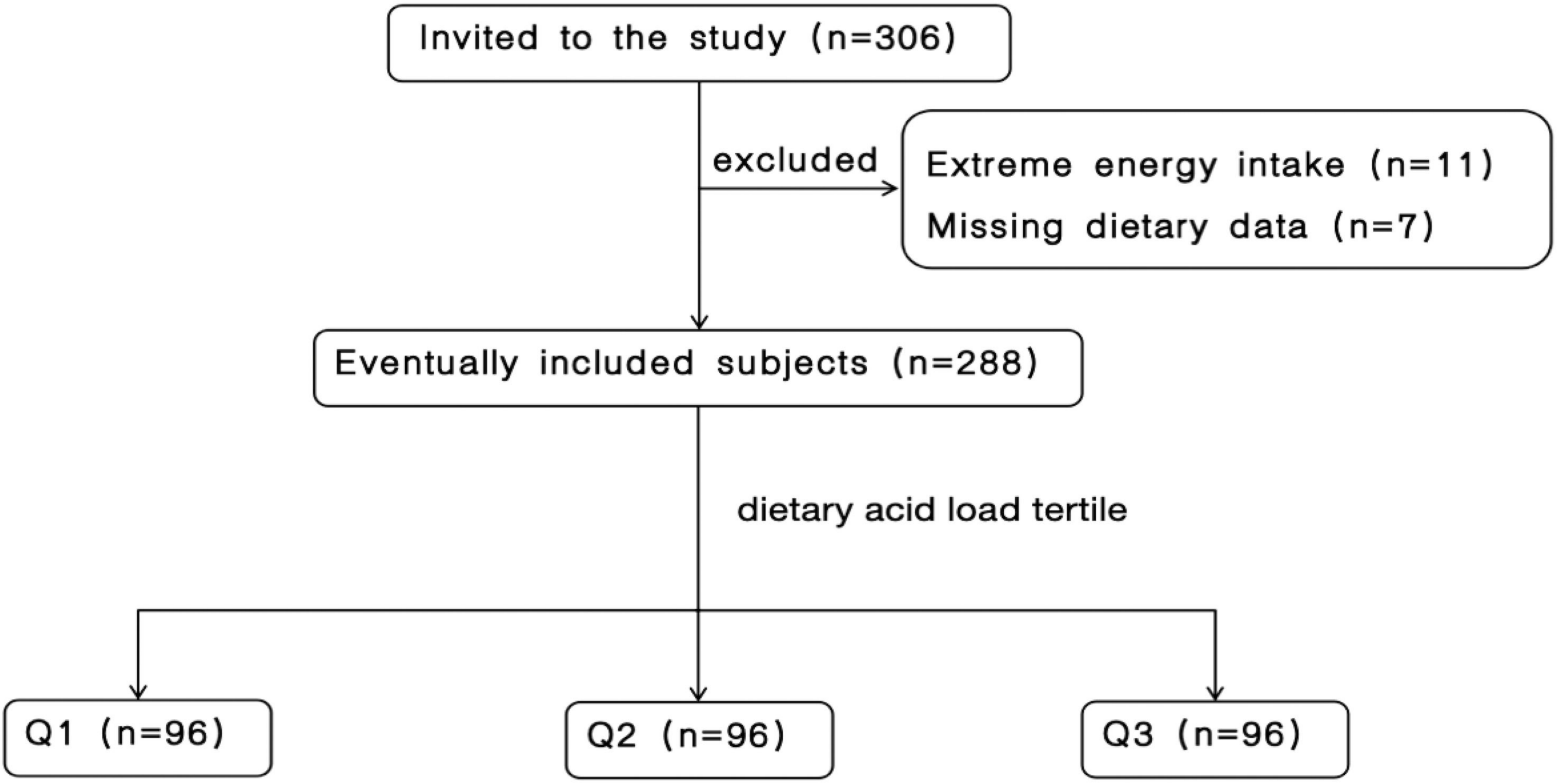
Flow chart of participant screening.

Initially, 306 patients with CKD were eligible for inclusion. After excluding 7 patients due to incomplete dietary information and 11 patients with extreme energy intake (< 600 or > 4000 kcal/day), a sum of 288 patients were qualified for participation. **Figure 1** shows the participant inclusion flowchart.

Among the DAL tertiles, PRAL, NEAP, DAL, TyG-BMI, TyG, TG/HDL-C, METS-IR, and BMI showed upward trends, and the differences were statistically significant (P < 0.001). Among the study participants, 141 (49%) had T2DM, and the difference across the tertiles was statistically significant (P = 0.008). The distributions of age, CKD course, and HTN among the DAL tertiles were not statistically significant (P or P-trend > 0.05). **Table 1** presents the characteristics of the patients.

**Table 1:** Baseline characteristics of study participants by categories of DAL.

Characteristics	N=288	Q1 (N=96)	Q2 (N=96)	Q3 (N=96)	P	P-trend
PRAL (mEq/day)	14.21 (5.60,21.25)	0.92 (-5.95,6.37)	14.40 (10.67,17.75)	23.99 (19.76,28.73)	<0.001	<0.001
NEAP (mEq/day)	66.37 (51.64,81.33)	44.88 (37.01,53.29)	66.85 (59.43,76.34)	84.64 (76.73,100.76)	<0.001	<0.001
DAL (mEq/day)	55.74 ± 14.50	40.45 ± 8.91	55.86 ± 3.45	70.94 ± 8.62	<0.001	<0.001
Age (years)	54 (41,61)	54.5 (43,61)	53 (38,62)	54 (42,60)	0.666	0.652
Gender						
Male, n (%)	185 (64.2%)	49 (51%)	52 (54.2%)	84 (87.6%)	<0.001	–
Female, n (%)	103 (35.8%)	47 (49%)	44 (45.8%)	12 (12.5%)		
BMI (kg/m²)	24.96 ± 3.63	24.3 ± 3.63	24.1 ± 3.62	26.47 ± 3.17	<0.001	<0.001
CKD course (months)	25.5 (12,83.25)	23 (11,60)	29 (12,93)	26.5 (12,96)	0.211	0.167
T2DM, n (%)	141 (49%)	44 (45.8%)	38 (39.6%)	59 (61.5)	0.008	–
Hypertension, n (%)	217 (75.3%)	68 (70.8%)	71 (74%)	78 (81.3%)	0.228	–
TyG	5.92 (5.51,6.41)	5.91 (5.46,6.33)	5.76 (5.40,6.34)	6.02 (5.71,6,59)	0.011	0.028
TyG-BMI	150.32 ± 31.71	145.33 ± 31.78	142.8 ± 32.20	162.82 ± 27.41	<0.001	<0.001
TG/HDL-C	3.77 (2.31,5.55)	3.22 (2.21,5.63)	2.92 (1.86,5.05)	4.14 (3.19,6.40)	0.001	0.009
METS-IR	38.89 (33.4,44.98)	37.81 (31.74,42.35)	36.14 (31.36,41.95)	42.1 (38,47.44)	<0.001	<0.001

PRAL, potential renal acid load; NEAP, net endogenous acid production; DAL, dietary acid load; BMI, body mass index; CKD, chronic kidney disease; T2DM, Type 2 Diabetes Mellitus. TyG, triglyceride and glucose index; TyG‐BMI, triglyceride and glucose index with body mass index;TG/HDL‐C, the ratio of triglycerides divided by high‐density lipoprotein cholesterol; METS‐IR, metabolic score for insulin resistance.

Among the DAL tertiles, patients with the highest DAL scores had significantly higher BUN, serum creatinine, serum cystatin C, uric acid, and FBG levels, all showing significant upward trends (P-trend < 0.05), while those assigned to the highest DAL category had significantly lower HDL-C levels (P = 0.006). However, there were no significant correlations between the DAL tertiles and WBC count, hemoglobin, total protein, haptoglobin, prealbumin, eGFR, 24-h urinary protein, total cholesterol, triglyceride, serum calcium, serum potassium, serum phosphorus, or LDL-C (P > 0.05). The clinical features are shown in **Table 2**.

**Table 2:** Comparison of clinical characteristics of study participants by categories of DAL.

Characteristics	N=288	Q1 (N=96)	Q2 (N=96)	Q3 (N=96)	P	P-trend
WBC (×10^9/L)	6.57 (5.19,8.03)	6.82 (4.95,8.65)	6.62 (5.23,8.07)	6.36 (5.58,7.72)	0.914	0.676
Hemoglobin (g/L)	118 (103,131)	116 (100.25,129.75)	115.26 (102,128.75)	122 (106.5,137.5)	0.091	0.067
Total protein (g/L)	58.5 (51.33,64.8)	58.6 (50.55,65.08)	57.4 (51.33,63.38)	59.35 (52.33,65.73)	0.527	0.929
Haptoglobin (mg/dl)	133.78 (85.58,160.75)	133.78 (92.53,164.5)	120.5 (69.5,154)	133.78 (98.1,164.5)	0.120	0.806
Prealbumin (mg/dl)	29.79 (24.43,33)	29 (23.83,32.75)	29.79 (24.7,32.5)	29.9 (24.85,34.28)	0.392	0.176
BUN (mmol/L)	8.11 (5.79,11.17)	7.46 (5.51,10.54)	8.05 (5.53,11.72)	9.27 (6.34,11.48)	0.061	0.019
Serum creatinine (umol/L)	116.25 (81.5,187.65)	109 (77.48,173.18)	105.55 (79.38,155.68)	147.6 (84.98,221.85)	0.054	0.032
eGFR (ml/min/1.73)	55.7 (30.66,88.08)	58.77 (36.64,90.55)	59.98 (33.22,89.34)	44.55 (27.92,83.61)	0.238	0.139
Cystatin C (mg/L)	1.78 (1.21,1.98)	1.65 (1.11,1.87)	1.68 (1.2,2)	1.78 (1.36,2.12)	0.101	0.041
24-hour urinary protein (g/24h)	2.01 (0.63,4.56)	1.48 (0.51,4.56)	2.24 (0.8,4.24)	2.14 (0.87,4.68)	0.250	0.096
Uric acid (umol/L)	375.65 (303.98,454.55)	358.9 (274.83,420.45)	375.2 (306.45,438.48)	388.65 (323.58,465.73)	0.021	0.005
Total cholesterol (mmol/L)	4.52 (3.83,5.44)	4.61 (3.75,5.49)	4.45 (3.8,5.44)	4.6 (3.88,5.37)	0.935	0.904
Triglyceride (mmol/L)	1.77 (1.21,2.43)	1.72 (1.2,2.31)	1.54 (1.11,2.31)	1.92 (1.45,2.64)	0.012	0.079
FBG (mmol/L)	4.79 (4.28,5.59)	4.59 (4.14,5.23)	4.8 (4.3,5.49)	4.89 (4.37,6.41)	0.074	0.023
Serum calcium (mmol/L)	2.19 (2.06,2.27)	2.21 (2.06,2.28)	2.19 (2.05,2.27)	2.16 (2.06,2.26)	0.424	0.191
Serum potassium (mmol/L)	3.89 (3.62,4.25)	3.9 (3.71,4.31)	3.87 (3.61,4.21)	3.88 (3.53,4.26)	0.401	0.180
Serum phosphorus (mmol/L)	1.26 (1.12,1.41)	1.27 (1.12,1.41)	1.28 (1.14,1.42)	1.26 (1.12,1.41)	0.913	0.779
HDL-C (mmol/L)	1.1 (0.91,1.33)	1.13 (0.92,1.5)	1.15 (0.95,1.43)	1.04 (0.87,1.18)	0.003	0.006
LDL-C (mmol/L)	2.69 (2.09,3.52)	2.73 (2.09,3.63)	2.61 (2.12,3.34)	2.71 (2.03,3.5)	0.874	0.859

WBC, white blood cell; BUN, blood urea nitrogen; eGFR, estimated glomerular filtration rate; FBG, fasting blood glucose; HDL-C, high-density lipoprotein cholesterol; LDL-C, low-density lipoprotein cholesterol.

Among the food groups, the DAL tertile was significantly positively correlated with meat and eggs (P < 0.05), while displaying a negative correlation with fruits and vegetables (P < 0.001). No significant statistical relationship was found between the consumption of grain or dairy product and the DAL tertile (P > 0.05). In terms of energy and macronutrient consumption, the DAL tertile was positively associated with protein, animal protein, monounsaturated fatty acids (MUFAs), saturated fatty acids, and fat intake (P < 0.05). Conversely, there was an inverse relationship with the intake of carbohydrate, plant protein, and fiber (P < 0.05). No significant association was found for polyunsaturated fatty acid (PUFA) or energy intakes across the DAL tertiles. Regarding dietary micronutrient intake, the DAL tertile was positively associated with phosphorus intake (P-trend < 0.05), but negatively associated with potassium, calcium, magnesium, and copper intake (P-trend <0.05). There was no significant correlation between the DAL tertile and sodium, iron, or zinc levels (P > 0.05). **Table 3** presents the nutritional intake data for the participants.

**Table 3:** Distribution of dietary intake in the study population.

Variables	N=288	Q1 (N=96)	Q2 (N=96)	Q3 (N=96)	P	P-trend
Food groups						
Grain (g/day)	335 (221.75,489.78)	360 (235.9,493.08)	316.05 (215.35,468.68)	320.2 (220.3,502.55)	0.352	0.497
Vegetables (g/day)	300 (178.5,435.9)	398.9 (237.38,586.5)	284.45 (198.43,377.5)	243.45 (141.45,329.8)	<0.001	<0.001
Fruits (g/day)	100 (0,220.88)	200 (40.55,400)	120 (0,200)	0 (0,150)	<0.001	<0.001
meat (g/day)	58.15 (10,140)	35 (0,95.48)	35 (6.25,100)	120.4 (50,184.38)	<0.001	<0.001
Eggs (g/day)	60 (0,61.2)	49.50 (0,60)	60 (0,84.28)	60 (0,90)	0.009	0.002
Dairy products (ml/day)	0 (0,250)	0 (0,237.5)	0 (0,250)	0 (0,250)	0.881	0.656
energy and Macronutrients						
Energy (kcal/day)	1,320.3 (1,027.03,1,644.79)	1,371.06 (1,031.2,1,631.2)	1,186.4 (954.85,1,524.49)	1,411.87 (1,142.26,1,953.51)	0.002	0.139
Carbohydrates (g/day)	213.99 (187.98,239.79)	223.26 (203.5,252.74)	215.63 (192.81,240.82)	196.47 (165.87,229.8)	<0.001	<0.001
Protein (g/day)	59.59 (53.31,68.4)	54.89 (50.57,59.62)	59.94 (54.57,66.74)	68.27 (59.07,81.77)	<0.001	<0.001
Plant protein (g/day)	31.71 (26.32,36.89)	32.97 (29.06,38.28)	31.05 (26.6,37.39)	31.51 (23.55,36.25)	<0.001	0.043
Animal protein (g/day)	28.78 (19,39.65)	22.8 (15.53,30.4)	27.91 (19.93,39.62)	37.48 (26.73,53.54)	0.127	<0.001
Fiber (g/day)	8.04 (5.46,11.02)	10.95 (8.11,13.99)	7.91 (6.18,10.05)	5.71 (3.63,8.12)	<0.001	<0.001
MUFA (mg/day)	11.57 (8.16,16.35)	10.20 (6.47,14.36)	11.43 (8.16,15.31)	13.45 (9.44,18.22)	0.004	0.001
PUFA (mg/day)	3.92 (2.57,5.26)	3.70 (2.34,4.95)	4.02 (2.65,5.34)	4.21 (2.76,6.05)	0.274	0.108
Saturated fat (mg/day)	12.93 (8.52,16.65)	11.37 (6.93,14.78)	12.97 (8.26,16.18)	14.82 (11.39,19.7)	<0.001	<0.001
Fat (g/day)	39.92 (29.94,48.28)	37.78 (28.12,45.6)	40.15 (29.94,47.71)	43.57 (32.81,53.99)	0.035	0.011
Micronutrients						
Sodium (mg/day)	1,023.1 (729.42,1,725.25)	1,018.54 (725.16,1,705.6)	1,008.12 (729.27,1,609.58)	1,050.09 (729.42,1,910.9)	0.642	0.374
Potassium (mg/day)	1,735.74 (1,418.71,2,151.91)	2,160.39 (1,767.6,2,462.92)	1,666.67 (1,406.79,1,953.21)	1,509.92 (1,255.68,1,824.45)	<0.001	<0.001
Calcium (mg/day)	374.19 (248.86,553.66)	429.77 (258.81,642.27)	353.71 (246.5,539.07)	358.17 (204.47,526.7)	0.028	0.010
Magnesium (mg/day)	264.51 (222.54,307.05)	293.92 (250.1,358.04)	257.13 (223.09,291.62)	233.16 (210.84,279.07)	<0.001	<0.001
Phosphorus (mg/day)	860.19 (758.4,1,011.7)	838.5 (739.84,1,006.34)	858.31 (759.11,953.6)	919.77 (791.08,1,098.93)	0.028	0.034
Iron (mg/day)	14.89 (11.9,18.08)	15.17 (12.38,18.78)	14.68 (11.9,17.15)	14.65 (11.53,17.56)	0.380	0.208
Copper (mg/day)	1.14 (0.87,1.49)	1.32 (0.99,1.81)	1.14 (0.91,1.33)	1.02 (0.78,1.22)	<0.001	<0.001
Zinc (mg/day)	7.30 (6.1,8.93)	7.45 (6.16,9.13)	7.05 (6.05,8.14)	7.43 (5.97,10.09)	0.268	0.764

MUFA, mono-unsaturated fatty acids; PUFA, polyunsaturated fatty acids.

There were no significant correlations between PRAL, NEAP, and the four non-insulin-based IR indices (P > 0.05). The DAL score was significantly associated with TyG-BMI (r = 0.251, P < 0.001) and METS-IR (r = 0.274, P < 0.001), but only weakly correlated with the TG/HDL-C ratio (r = 0.14, P = 0.018). **Table 4** presents the correlations between dietary acid-base load scores and the four non-insulin-based IR indices.

**Table 4:** Correlation analysis between dietary acid-base load scores and four types of non-insulin resistance.

indices	PRAL		NEAP		DAL	
	r	P	r	p	r	p
TyG	0.028	0.636	-0.026	0.656	0.104	0.078
TyG-BMI	0.086	0.148	0.017	0.779	0.251	<0.001
TG/HDL-C	0.042	0.474	-0.015	0.804	0.14	0.018
METS-IR	0.076	0.196	0.008	0.897	0.274	<0.001

**Table 5** presents the association between the DAL score and four types of non-insulin resistance in CKD patients. In the rough model, model 1, and model 2, the DAL score was associated with both TyG -BMI (β = 0.549, P < 0.001; β = 0.437, P = 0.001; β = 0.336, P = 0.008, respectively) and METS-IR (β = 0.161, P < 0.001; β = 0.118, P = 0.001; β = 0.091, P = 0.007, respectively). There was no significant correlation, however, between the DAL score and the TyG or TG/HDL-C (P > 0.05).

**Table 5:** Association between DAL score and four types of non-insulin resistance in CKD patients.

indices	β	SE	P
TyG			
Crude	0.004	0.003	0.179
Model1	0.002	0.003	0.399
Model 2	0.001	0.003	0.825
TyG-BMI			
Crude	0.549	0.125	<0.001
Model1	0.437	0.128	0.001
Model 2	0.336	0.127	0.008
TG/HDL-C			
Crude	0.022	0.016	0.154
Model1	0.011	0.016	0.493
Model 2	0.005	0.016	0.781
METS-IR			
Crude	0.161	0.033	<0.001
Model1	0.118	0.034	0.001
Model 2	0.091	0.033	0.007

Crude: unadjusted; Model1: adjusted for gender, age, BMI and energy intake; Model 2: adjusted for gender, age, BMI, energy intake, Hypertension, Type 2 Diabetes Mellitus and eGFR. DAL, dietary acid load; NEAP net endoge-nous acid production; PRAL, potential renal acid load; TyG, triglyceride and glucose index; TyG‐BMI, triglyceride and glucose index with body mass index;TG/HDL‐C, the ratio of triglycer-ides divided by high‐density lipoprotein cholesterol; METS‐IR, metabolic score for insulin re-sistance.

## 4 Discussion

In terms of energy and macronutrients, the study found that patients who were in the highest tertile of DAL had a higher intake of protein, animal protein, MUFAs, saturated fat, fat, and phosphorus, but a lower intake of carbohydrates, plant protein, dietary fiber, potassium, calcium, magnesium, and copper. The sources and types of dietary protein are essential for preventing disease. Some studies have shown that the total protein and animal protein can increase the risk of T2DM, whereas plant protein can reduce it (29, 30). Several prospective studies indicate that total MUFA intake is negatively related to the probability of T2DM (31, 32). Another study showed that a higher seafood source of omega 3 PUFAs is correlated with a lower risk of developing CKD (33). Whether fatty acids are a risk or protective factor for T2DM remains controversial, possibly because of differences in food sources and carbon chain lengths (34, 35).

In this study, DAL scores were significantly connected with TyG-BMI and METS-IR scores, but were only mildly correlated with TG/HDL-C. The four non-insulin-based IR markers, calculated from human biochemical indicators, are economical, simple and convenient. Of these four measures, TyG combined FPG and lipid levels, while the TG/HDL-C ratio is a key component of hyperlipidemia. TyG-BMI and METS-IR included not only blood lipids and FBG, but also BMI, which is important because obesity induces chronic inflammation leading to IR and metabolic disorders (36). Researches indicates that being overweight heightens the likelihood of CKD pathogenesis, deterioration of kidney function, and progression to end-stage kidney disease (37, 38). Mechanistically, IR leads to renal hemodynamic changes, tubular dysfunction, chronic inflammation and fibrosis (39, 40).

After adjusting for sex, age, energy, HTN, diabetes, and eGFR, multiple linear regression analysis showed that DAL score was associated with TyG-BMI (β = 0.336, *P* = 0.008) and METS-IR (β = 0.091, *P* = 0.007). Our research aligns with previous studies, suggests that TyG-BMI and METS-IR serve as more effective and dependable measures for evaluating IR and forecasting cardiovascular outcomes (9, 41, 42). Results from a Korean cohort study indicated that participants in the highest PRAL quartile had a 1.30 times higher risk of IR compared with the lowest quartile; Similar risk estimates have been observed for NEAP scores (18). Studies in Japan have shown that a higher dietary acid-base load (PRAL and NEAP scores) is connected with IR (43).Studies conducted in Denmark have shown that higher PRAL scores are associated with IR (44). Similarly, in this study, the DAL score was significantly positively correlated with TyG-BMI and METS-IR. A prospective study of 70–71 years old men in Swedish showed that the PRAL and NEAP scores were not associated with insulin sensitivity or β-cell function, in contrast to our results. This discrepancy may be due to the inconsistent age and sex of the study population, as we included both male and female participants over 18 years of age. Multiple mechanisms have been proposed to explain the associations between DAL and IR. Metabolic acidosis not only increases the secretion of cortisol and glucocorticoids (45), but also inhibits the level of adipokine (46), thereby increasing IR. In addition, the acidic environment may reduce insulin sensitivity by affecting insulin-like growth factor I (IGF-I) (47, 48). Reduced interstitial pH impairs insulin-receptor binding, leading to IR (49, 50).The study showed that metabolic acidosis from diet altered insulin secretion and signaling (51).

IR is associated with the occurrence and progression of CKD, which is a serious threat to public health because of its high prevalence, poor prognosis, and high mortality. The intake of acidic and alkaline foods affects the body’s acid-base balance, which significantly affects the risk of CKD progression (52). We assessed the connection the between dietary acid-base load and non-insulin-based IR markers in patients with CKD.

Both PRAL and NEAP scores were derived from protein and micronutrients. For micronutrients, the PRAL score includes phosphorus, potassium, magnesium and calcium (12); the NEAP score is determined by potassium intake (13); and the DAL score is calculated based on the PRAL score and body surface area (14). For these metrics, a higher score indicates that the food has a higher acidogenic potential. As each measure has a different formula, limitations, and strengths, the use of all three dietary acid-base load measures may provide more reliable results than any measure alone. The food group associated with the highest DAL tertile in this study was distinguished by increased intake of meat and eggs, but decreased consumption of vegetables and fruits. With respect to the body acid load, plant-based dietary patterns are thought to reduce, while animal-based dietary patterns are thought to increase. Vegetables and fruits contain excellent antioxidants, which can eliminate free radicals, prevent oxidative stress, and protect cells and structures from oxidative damage (53). A rise in the amount of the consumption of fruits and vegetables has long been linked to benefits against cancers, diabetes, neurodegenerative diseases, and cardiovascular diseases (54). However, meat consumption, and synthetic meat in particular, is positively connected with the production of proinflammatory substances (55).

To our knowledge, this study is the first to examine the relationship between dietary acid load and the four non-insulin-based IR indices in patients with CKD. Previous studies did not consider diet as a confounding factor, and adjustments were made for energy in the model used in this study. Nevertheless, this study has some certain limitations. First, this study was cross-sectional and unable to assess causality, a prospective cohort study is recommended for further validation studies. The diversity and representativeness of the sample may be limited. Subsequent studies are recommended to validate the findings in groups of different regions and ethnicities. Second, the 24-h dietary recall is potentially susceptible to recall bias. Future studies may employ 3-day dietary records or food frequency questionnaires to more accurately assess dietary intake. Third, dietary supplements were not considered. Other confounding factors that were not adjusted for may have influenced our study results, such as physical activity and medication use including statins and fibrates. According to our results, the DAL score was significantly positively associated with TyG-BMI and METS-IR after adjusting for possible confounders. This indicates that reducing dietary acid load is a healthy dietary habit that may help prevent IR-related diseases with CKD.

## 5 Conclusions

According to our current study, the patients with the highest DAL scores had the highest TyG, TyG-BMI, TG/HDL-C ratio, and METS-IR. After adjusting for possible confounding factors, the DAL score exhibited a significant positive correlation with both TyG-BMI and METS-IR. Reducing dietary acid load by consuming more fruits and vegetables and limiting excessive intake of meat is beneficial for some diseases caused by IR.

## Data Availability

The original contributions presented in the study are included in the article/Supplementary Material. Further inquiries can be directed to the corresponding authors.
